# An analysis of COVID-19 information sources

**DOI:** 10.1186/s40545-022-00446-8

**Published:** 2022-08-17

**Authors:** Belachew Umeta, Temesgen Mulugeta, Girma Mamo, Sintayehu Alemu, Nimona Berhanu, Gudina Milkessa, Birhanu Mengistu, Tsegaye Melaku

**Affiliations:** 1 School of Pharmacy, Institute of Health, Jimma University, P.O. Box: 378, Jimma, Oromia Ethiopia; 2 Department of Ophthalmology, School of Medicine, Institute of Health, Jimma University, Jimma, Oromia Ethiopia; 3 Department of Anesthesia, Institute of Health, Jimma University, Jimma, Oromia Ethiopia

**Keywords:** COVID-19, COVID-19 information source, COVID-19 information gaps, COVID-19 information trust

## Abstract

**Background:**

The COVID-19 pandemic has brought new situations that require the effective delivery of health information across the world and it’s important to offer clear, consistent, and credible information on the pandemic to mitigate and control the outbreak.

**Aim:**

To assess COVID-19 information source, need and trust among the rural community of southwest Ethiopia.

**Methods:**

A community-based cross-sectional study design was conducted among 634 rural communities of southwest Ethiopia. The data were collected by interviewing individuals from selected households and analyzed by SPSS version 26. A multivariable logistic regression model was used to assess factors affecting information needs.

**Results:**

Radio 484 (76.3%) was mostly used as a source of information for COVID-19, and government 404 (63.7%) and health professionals 345 (57.7%) were trusted. However, only 10 (1.6%) of the participants acquired information from health professionals. Around 395 (62.3%) of the participants needed additional information on COVID-19. Around 230 (58.2%) and 186 (47.1%) of the participants required additional information on cause and sign and symptoms, respectively. Age of < 45 years old (AOR: 2.11, 95% CI: 1.43, 3.12, *P* < 0.001), and absence of formal education (AOR: 2.00, 95% CI: 1.35, 2.95, P: 0.001) were factors positively affecting the information needs of the participants on COVID-19. Church goers (AOR: 3.24; 95% CI: 2.03, 5.19; *P* < 0.001), television (AOR: 2.39; 95% CI: 1.63, 3.49; *P* < 0.001) and social media users (AOR: 4.52; 95% CI: 2.26, 9.04; *P* < 0.001) as source of information required additional information on COVID-19, and the participants that trusted social media (AOR: 2.52; 95% CI: 1.64, 3.87; *P* < 0.001) and friends/relatives (AOR: 2.95: 95% CI: 1.51, 5.76; *P* < 0.001) were also required additional information on COVID-19.

**Conclusions:**

The popular sources of COVID-19-related information were radio and television. The participants trusted the government and health professionals on COVID-19. However, less than 2% of the participants had information from health professionals. The majority of the participants wanted to learn more about COVID-19. The areas the participants required additional information include cause and signs and symptoms. Age, educational status, trust in social media, trust in friends, using the church, television and social media as a source were factors associated with information needs.

## Background

The COVID-19 pandemic has brought new situations that require the effective delivery of health information across the world. A previous study has indicated that media hype has induced panic and confusion among the public [1]. This is compounded by the misinformation and disinformation circulating on social media during the pandemic. During the pandemic, people place a greater reliance on the government and health officials to handle the issue [2]. It is critical to offer clear, consistent, and credible information on the pandemic to mitigate and control the outbreak [3]. Timeliness and precision, while challenging to achieve and assess, are critical for both the public and the scientific community in reducing and managing the pandemic. [3]

The internet is the most common source of information about the etiologies and treatment models among the general public [4]. According to recent research of 21 countries, the amount of Google searches for "wash hands" increased as the COVID-19 spread slowed [5]. On the other hand, misinformation about COVID-19 has been circulating on the internet, particularly on social media [6, 7]. In addition to the internet, traditional media are also important sources of information during disease outbreaks [8]. However, repeated media exposure to crisis-related information elevates anxiety and stress responses among people [9]. Medical staff and laypeople, such as friends, family members, and coworkers, may also provide information on COVID-19 to the general public. Health practitioners can better educate the public by studying the elements that influence such information sources. By building transparent and effective information-delivery methods, their audience's self-confidence in dealing with the outbreak can be improved [10].

Measures to limit the pandemic have caused economic and social disturbances, with significant increases in unemployment, poverty, and psychological suffering [11, 12]. As a result, authorities face the difficulty of persuading the public that these steps are justified and required in order to avoid a possibly worse disaster. When establishing messaging, designing and implementing risk communication strategies, the way information is formulated, the channels through which it is disseminated, and the population targeted must be taken into account [13].

In today's information era, there are an ever-increasing number of sources for health-related information, and the public has shifted away from relying solely on major news channels and toward other sources of information, such as social media [14, 15]. In the past, mainstream media outlets such as television and newspapers have been important sources of information during infectious disease outbreaks [16, 19].

Ethiopia has been hit by the pandemic when the country's political and security situations are particularly perilous [20]. The government has taken several measures to counter COVID-19. Borders were close, and so were the schools and the nightclubs. Retired and in-training medical personnel were called, and all people entering Ethiopia from abroad were subjected to mandatory quarantine during the pandemic era. Ethiopian Airlines has halted flights to 80 destinations around the world [21]. The accuracy, completeness, and verification of information sources vary, particularly in a highly polarized political climate when antiscientific rhetoric and political bias may underpin reporting by numerous media channels [15].

According to a survey of Jordanian university students (2020), the internet (77.1%) was the most common source of information about COVID-19, including electronic news websites and social media like Twitter, Facebook, YouTube, Instagram, Snapchat, and WhatsApp, followed by mass media (67.6%) like television, newspapers, magazines, and radio, and scientific websites and articles (24.2%). Only 7.00% of the participants got information from friends and relatives [22]. In Taiwan, a Facebook poll found that the internet was the leading source of COVID-19 information for a substantial percentage of users, followed by traditional media, family members, coworkers, friends, formal lessons, and medical personnel [10]. As a result, the current study aims to assess COVID-19 information source, need, and trust among the rural community of Southwest Ethiopia.

## Materials and methods

### Study design, setting and period

A community-based cross-sectional study design was employed in the rural community of Jimma Zone, Southwest Ethiopia. Jimma town is the capital city of Jimma zone. The town is located 357 km Southwest of Addis Ababa, the capital city of Ethiopia. Currently, the Zone has 22 Woredas and 2 administrative towns. According to the 2007 census, the Zone had a total population of 2,486,155, of which 1,250,527 were men [43]. The total households counted were 521,506. The study was conducted from March–April 2021.

### Study population

The participants were selected from households in the Jimma zone. Adults > 18 years old and willing to participate were included. Individuals who were mentally ill and incapable of communicating were excluded.

### Sample size and sampling technique

To calculate the sample size, a single population proportion formula (*n* = *Z*2*P* (1 − *P*)/*d*2) was used. Where, *P* = 50% (prevalence of information void in the community) and *Z* = 1.96 and margin of error 5%. Based on the above assumption, the total sample size was 384. Due to the multistage sampling technique, a design effect of 1.5 was considered. Since the COVID-19 case is new, the community response rate might be reduced, and we consider a non-response rate of 10%. Finally, the total sample size included in the study was 634.

A lottery method was used to select Woredas. A proportional allocation was used to determine the sample size of respondents from each Woredas. Finally, the participants were selected using a systematic random sampling procedure.

### Data collection tools and technique

A pretested semi-structured questionnaire was used for the data collection from the selected households. The pretest was conducted on 5% of non-sampled individuals to check the reliability and clarity of the questionnaires. The questionnaire was prepared in English by reviewing different literature [23, 24]. The questionnaires consists of four subsections (sociodemographic characteristics of participants, community information source and trust on COVID-19, community information need on COVID-19 and opinion of community on government preparedness to control the pandemic).

The questionnaire consists of sociodemographic characteristics: sex, age, religion, marital status, occupation, educational status and family size.

Community information source and trust: common source of information on COVID-19 [radio, relatives, church, television, health professionals, internet based sources, mobile texting, megaphone, newspapers, other (please specify)]; who do you trust most on COVID-19 information [government, health professionals, social medias, religious leaders, friends/relatives, traditional healers, don’t know who to trust, No one, other (please specify)].

Community information needs: We asked the participants a question stating “do you need additional information on COVID-19?” and if their response was “yes”, we followed a question “on which area you need additional information [cause, signs and symptoms of the disease, ways to prevent the disease/prevention methods, medical care and treatment options, other (please specify)]?”.

Opinion of community on government preparedness to control the pandemic: In your opinion, do you agree with government preparedness to control the pandemic? In your opinion, do you agree that the closure of schools and workplaces will help to control the pandemic? In your opinion, do you agree with the state of emergency launched by the government to control the pandemic? In your opinion, is the effort of the government to control the pandemic good?

The data collection was conducted by face-to-face interviews by trained data collectors on survey document. The interview lasts from 15 to 25 min. To ensure the anonymity of the data, no unique identifier was included on the survey document and the data collection sheet was coded. During data collection, the data collectors were maintained at least two meters (2 m) distance from the respondents, and the use of a facemask was mandatory.

### Data processing and analysis

The data were checked for completeness and consistency before entering into a computer. The data were entered into Epi-data version 3.1 and exported to SPSS version 26 for analysis. Descriptive analysis like frequency, percentage, and means (SD) was calculated. All variables having a *P*-value of < 0.25 in the univariable logistic regression analysis were entered into multiple variable logistic regressions to identify the predictors. A *P*-value of < 0.05 was used to declare statistical association.

## Results

### Sociodemographic characteristics of participants

More than half of the participants 337 (53.2%) were male, and the majority 475 (74.9%) were Muslims. The participants' average age (SD) was 38.58 (16.99). The majority of the participants 524 (82.6%) were married, and roughly 229 (36%) had no formal education. The average family size (SD) was 4.75 (1.83) (Table 1).

**Table 1:** Sociodemographic characteristics of participants

Characteristics	Frequency (%)
Sex	
Male	337 (53.2)
Female	297 (46.8)
Age in years (mean ± SD)	38.11 ± 11.34
Religion	
Muslims	475 (74.9)
Orthodox	117 (18.5)
Protestant	42 (6.6)
Marital status	
Single	71 (11.2)
Married	524 (82.6)
Divorced	10 (1.6)
Widowed	29 (4.6)
Educational status	
No formal education	229 (36.1)
Some primary education	163 (25.7)
Completed primary education	84 (13.2)
Completed secondary education	59 (9.3)
Diploma	52 (8.2)
Bachelors	47 (7.4)
Occupation	
Government	96 (15.10)
Self employed	190 (30.00)
Farmers	248 (39.10)
Others^a^	100 (15.8)
Family size (mean ± SD)	4.75 ± 1.83

*SD* standard deviations

^a^Merchant, housewife, student, driver, daily labor, religious leader, no work

### Community information sources and trust on COVID-19

The community used Radio 484 (76.3%), television 366 (57.7%), church 159 (25.1%), and mobile texting 144 (22.7%) as sources for COVID-19 information (Table 2).

**Table 2:** Community information source and trust about COVID-19

	Frequency (%)
Common source of information about COVID-19	
Radio	484 (76.3)
Relatives	143 (22.6)
Church	159 (25.1)
Television	366 (57.7)
Health professionals	10 (1.6)
Internet based sources	69 (10.9)
Mobile texting by government	144 (22.7)
Megaphone	143 (22.6)
Newspapers	20 (3.2)
Who do you trust on COVID-19 information flow?	
Government	404 (63.7)
Health professionals	345 (54.4)
Social medias	148 (23.3)
Religious leaders	136 (21.5)
Friends/relatives	66 (10.4)
Traditional healers	16 (2.5)
Don’t know who to trust	4 (0.6)
No one	50 (7.9)

The majority, 404 (63.7%) and 345 (54.4%) of them, trust information delivered by government and health professionals, respectively. Around 148 (23%) of the community trust information delivered through social Medias (Table 2).

### Community information needs on COVID-19 in Jimma Zone

The majority of the participants, 395 (62.3%), required additional information on COVID-19. The cause 230 (58.23%) and signs and symptoms 186 (47.09%) were the common areas in which the community required additional information on COVID-19. In addition, the community also required additional information on whether or not COVID-19 was man-made or not, why the disease is serious in pregnant mother, what was the fate of patient after recovery (Table 3).

**Table 3:** Community information gaps about COVID-19

Community information gaps on COVID-19 (*n* = 643)	Frequency (%)
Do you need additional information on COVID-19?	
Yes	395 (62.3)
No	239 (37.7)
On which area you need additional information about COVID-19? (*n* = 395)^b^	
Cause	230 (58.2)
Sign and symptoms	186 (47.1)
Ways of prevention	176 (44.6)
Medical care and treatment options	150 (38.0)
Others^a^	9 (2.3)

^a^Is COVID-19 man-made or not? Why the disease is serious in pregnant mothers? After recovery, what was the fate of patients in the future? How to give homecare to COVID-19 patients? How did it come to our country? About the vaccine? About the variant of the virus? Why does it affect patients with co-morbid conditions? Why awareness creation campaign is stopped?

^b^Percentage addition might be greater than hundred as more than one answer is possible

### Opinion of participants on government preparedness

More than half, 342 (53.9%) and 370 (58.4%) of the participants believed in government preparedness to control the pandemic and agreed that the closure of schools and workplaces would help in controlling the pandemic, respectively. Around 346 (54.6%) of the participants reported launching a state of emergency in the country would help in controlling the pandemic (Table 4).

**Table 4:** Participants opinion on government effort and preparedness to control the pandemic

Opinion of participants on government preparedness	Frequency (%)
Do you believe in the government preparedness for the control of the pandemic?	
Yes	342 (53.9)
No	200 (31.5)
I don’t know	92 (14.5)
Do you agree the closure of school and work place will help to control the pandemic?	
Yes	370 (58.4)
No	187 (29.5)
I don’t know	77 (12.1)
Is that good to launch the state of emergency by the government to control the pandemic?	
Yes	346 (54.6)
No	208 (32.8)
I don’t know	80 (12.6)
Are you satisfied with government efforts to control the pandemic?	
Yes	339 (53.5)
No	231 (36.4)
I don’t know	64 (10.1)

### Determinants of information need on COVID-19 in rural community of Southwest Ethiopia

Different sociodemographic factors were associated with information need of the community on COVID-19. Participants younger than 45 years old were 2.22 × in need of additional information on COVID-19 (95% CI: 1.43, 3.12; *P* ≤ 0.001). In addition, participants who did not have formal education were 2.00 × in need of additional information (95% CI: 1.35, 2.95; *P* ≤ 0.001) (Table 5).

**Table 5:** Sociodemographic characteristics of the participants and the need for COVID-19 related information

Sociodemographic characteristics	Information needs		COR	*P*-value	AOR	95% CI	*P*-value
	Yes	No					
Gender							
Male	217	120	1.21	0.248	1.3	0.93, 1.83	0.131
Female	178	119	1		1		
Age							
< 45 years old	242	101	1.87	< 0.001	2.11	1.43, 3.12	< 0.001^#^
≥ 45 years old	153	138	1		1		
Religion							
Muslims	311	164	1.63	0.005	1.64	1.12, 2.39	0.011^#^
Others^a^	84	75	1		1		
Marital status							
Single	53	18	1.90	0.025	1.79	0.99, 3.23	0.052
Married	342	221	1		1		
Educational status							
No formal education	154	75	0.72	0.054	2.00	1.35, 2.95	< 0.001^#^
Primary education and above	241	164	1		1		
Family size							
< 5	278	150	1.41	0.048	1.34	0.93, 1.92	0.118
≥ 5	117	89	1		1		

*AOR* adjusted odds ratio, *CI* confidence interval, *COR* crude odds ration

^a^Orthodox, protestant

^#^Statistically significant at *P*-value of < 0.05

### Information sources and community’s need for additional information on COVID-19

The community that used places of religious worship as the source was 3.27 times in need of additional information (95% CI: 2.03, 5.19; *P* < 0.001) on COVID-19. In addition, the community that used Social Media as a source for COVID-19 was also 4.6 times in need of additional information (95% CI: 2.26, 9.04; *P* < 0.001) on COVID-19 (Table 6).

**Table 6:** Information sources and community’s need for additional information on COVID-19

Information sources	Information need		COR	*P*-value	AOR	95% CI	*P*-value
	Yes	No					
Radio							
Yes	318	166	1.82	0.002	1.28	0.85, 1.93	0.239
No	77	73	1		1		
Relatives							
Yes	98	45	1.42	0.082	1.14	0.73, 1.77	0.577
No	297	194	1		1		
Church							
Yes	131	28	3.74	< 0.001	3.24	2.03, 5.19	< 0.001*
No	264	211	1		1		
Television							
Yes	197	169	1		1		
No	198	70	2.43	< 0.001	2.39	1.63, 3.49	< 0.001*
Health professionals							
Yes	9	1	5.55	0.105	3.13	0.34, 29.17	0.316
No	386	238	1		1		
Social media							
Yes	58	11	3.57	< 0.001	4.52	2.26, 9.04	< 0.001*
No	337	228	1		1		
Mobile texting							
Yes	84	60	1	0.264			
No	311	179	1.24				
Megaphone							
Yes	94	49	1.21	0.336			
No	301	190	1				
Newspaper							
Yes	16	4	2.48	0.108	2.09	0.62, 7.04	0.233
No	379	235	1		1		

### Community’s information trust and needs for additional information on COVID-19

The community that trusted social Media had 2.52 times in need of additional information (95% CI: 1.64, 3.87; *P* < 0.001) on COVID-19. In addition, the community that trusted their relatives/friends was 2.95 times in need of additional information (95% CI: 1.51, 5.76; *P* 0.002) on COVID-19 (Table 7).

**Table 7:** Information trust of the community and the need for additional information on COVID-19

Information trust	Information need		COR	*P*-value	AOR	95% CI	*P*-value
	Yes	No					
Government							
Yes	265	139	1.47	0.024	1.41	1.00, 1.99	0.050
No	130	100	1		1		
Health professional							
Yes	229	116	1.46	0.021	1.38	0.99, 1.94	0.060
No	166	123	1		1		
Social media							
Yes	113	35	2.34	< 0.001	2.52	1.64, 3.87	< 0.001*
No	282	204	1		1		
Religious leaders							
Yes	98	38	1.75	0.009	1.38	0.89. 2.13	0.152
No	297	201	1		1		
Friends/relatives							
Yes	54	12	3.00	0.001	2.95	1.51, 5.76	0.002*
No	341	227	1		1		
Traditional medicine practitioners							
Yes	10	6	1.01	0.987			
No	385	233	1				

*AOR* adjusted odd ratio, *CI* confidence interval, *COR* crude odd ration

*Statistically significant at *P*-value of < 0.05

## Discussion

This community-level study was conducted in Southwest Ethiopia to assess community information sources for COVID-19, trust, and need for COVID-19 information. The study identified the community’s most common sources of COVID-19-related information were radio and television. Approximately two-thirds of the community trusted government information. Although only 2% of the community received information from health professionals, more than half of the community trusted them for COVID-19-related information. Most of those who took part said they wanted additional information on COVID-19. The top priority areas for additional information were causes, signs and symptoms, and prevention techniques. Age, religion, educational status, and usage of the church, television, and social media as a source of information, as well as trust in the contents of social media and friends/relatives, were the variables associated with community needs for additional information on COVID-19.

In this study, radio (76.3%) was the predominant source of information for COVID-19 among rural communities. In contrast, a study from Gondar city, Northwest Ethiopia reported that television (67.1%) was the number one source of information for COVID-19 [25]. Similarly, a finding from the residents of Addis Ababa reported that government-owned Media were the primary source of information for COVID-19 [26]. The difference might be related to the type of included participants. However, an online survey from Indonesia reported that social media (Facebook and Instagram) was the first information source for COVID-19 [27]. In Jordan, the internet (77.1%), including electronic news websites, was reported to be the primary source of information on COVID-19 [22]. We might relate the discrepancy between the studies to the age of the participants, the educational status of the participants, setting of the studies, and mode of data collection.

Our findings revealed that the majority (63.7%) of the community had trust in information delivered by the government on COVID-19, and less than one-quarter trusted social media. Similarly, in the US, government information sources were the most trusted among the public, and less than one-third of the participants trusted social media concerning COVID-19 information [28]. This might be related to risk perceptions towards coronavirus being lower when the community trusted the information delivered by the government. The implementation of lockdown measures might also increase trust in the government. Higher compliance rates and reduced death rates may be linked to trust [29]. However, during the Ebola pandemic in Liberia in 2018, a study found that community trust in non-governmental organizations (NGOs) was stronger than in government [30]. People seek information from various resources to update their knowledge and become more prepared in the face of COVID-19 [31]. Our result showed that 62.3% of the rural communities needed additional information about COVID-19. Similarly, 71.1% of health professionals at the University of Gondar Teaching and Referral Hospital have sought information on the COVID-19 pandemic [32]. In the current study, out of the respondents who needed additional information on COVID-19, the frequent area of topics mentioned for additional information was the cause (58.23%), followed by signs and symptoms (47.09%) and prevention methods (44.56%). Similarly, an online survey conducted in Indonesia during the pandemic showed that more than 78% of the respondents sought additional information on how to prevent the virus, and about 65% sought COVID-19 transmission in Indonesia, cause, treatment and symptoms [27]. The higher need for additional information on COVID-19 might indicate the inefficient delivery of information to the population. Regarding the respondents’ opinions on government preparedness, about 54.0% believe in government preparedness to control the pandemic. And a similar percentage of the respondents believed that launching a state of emergency would be good to control the pandemic. A qualitative study from Pakistan reported that training should be organized for communities to ensure better preparedness. Community members also suggested that the government should ensure strict compliance with standard operating procedures (SOPs) through regulatory reforms [33]. This study may be supported by a study conducted in Addis Ababa Ethiopia, the capital city of the country where 70% of service providers have made hand-washing facilities available [26]. Furthermore, our finding indicated that about half (53.5%) of the communities were satisfied with government efforts to control the pandemic. A multicenter study conducted in 14 countries by Chen et al. showed people pay strong attention to the results of their governments’ battle against COVID-19 (number of confirmed cases and deaths per million populations) rather than to what policies they initiate. Country-based comparable findings were reported from Spain (54.0%) and Japan (55.0%). However, relatively higher than the USA (47.0%) and the UK (46.0%) [34]. Similarly, a study conducted in Indonesia reported lower satisfaction of communities with the local government's performances, which include: anticipation, early detection, containment, control and mitigation, and elimination [35].

As per the current study, respondents younger than 45 years were more likely to require additional information about COVID-19. This finding might be related to the fact that these age groups were daily laborers and might not have adequate time to listen, read and participate in community awareness campaign programs organized by the healthcare professionals. Our result also showed that religious individuals were more likely to require additional information about COVID-19. In support of this finding, a study from Northeastern Ethiopia reported that 47.8% of the respondents believed that COVID-19 emerged because of the sin of human beings [36]. Believers conflict with authorities’ warnings that gatherings must be limited to combat the spread of the virus [37]. Moreover, illiterate respondents were more likely to require additional COVID-19 information. A study done in Germany reported there was a significant association between low educational levels and higher perceived COVID-19 severity [38]. This is because sufficient health literacy is an essential prerequisite for finding, understanding, appraising, and applying health recommendations, particularly in a situation where there is a rapid spread of a huge amount of information [39].

Concerning sources of information for COVID-19, our findings identified respondents who obtained COVID-19 related information from religious worship and social media were more likely to require additional information on COVID-19. Similar to these findings, Hsing-YingHo et al. reported that COVID-19-related information from internet/traditional media and friends was associated with higher levels of public worry. In contrast, participants who received information from academic courses had lower past worry and anticipated worry [40]. This might be related to the quantity of information delivered and the credibility of information contained.

In addition, our findings showed that trust in information sources such as social media and friends and relatives was associated with the need for further COVID-19 information. Similarly, Maykrantz et al. reported that worry about contracting COVID-19 is related to trust in formal information sources (government and media), but not too informal ones (interpersonal) [41]. A study conducted in the USA also reported that public trust in social networks (e.g., Facebook and Twitter) was negatively associated with knowledge and adherence to social distancing [28]. This might be related to the huge COVID-19 information circulating on the social media or the internet had not been officially certified by HONcode [42].

Conclusions:- The popular sources of COVID-19-related information were radio and television. The participants trusted the government and health professionals on COVID-19. However, < 2% of the participants had information from health professionals. The majority of the participants wanted to learn more about COVID-19. The areas the participants required additional information include cause and signs and symptoms. Age, religion, educational status, trust in social media, trust in friends, and using the church, television and social media as a source were associated with information needs.

## Data Availability

The datasets used and/or analyzed during the current study are available from the corresponding author on reasonable request.
